# Exploring the associations of depressive symptoms with healthy eating self-efficacy over time amongst women in the READI cohort study

**DOI:** 10.1186/s12966-021-01233-5

**Published:** 2021-12-18

**Authors:** Rachelle Opie, Gavin Abbott, David Crawford, Kylie Ball

**Affiliations:** Institute for Physical Activity and Nutrition, School of Exercise and Nutrition Sciences, Faculty of Health, Deakin University, 221 Burwood Highway, Burwood, VIC 3125 Australia

**Keywords:** Healthy eating, Diet, Depression, Depressive symptoms, Self-efficacy, Women, Socioeconomic disadvantage

## Abstract

**Background:**

There is growing evidence that diet is associated with both depressive symptoms and clinical depression, likely through biological mechanisms. However, it is also plausible that depression impacts diet, for example by impairing the personal drivers of healthy eating behaviors such as self-efficacy. This study is one of the first to explore the association of depressive symptoms with healthy eating self-efficacy over time.

**Methods:**

Data was drawn from the Resilience for Eating and Activity Despite Inequality (READI) longitudinal study, a prospective cohort study of socioeconomically disadvantaged Australian women. This analysis includes a sub-sample of 1264 women. Linear mixed models, with random intercepts for suburb of residence, were performed to explore the relationships between total healthy eating self-efficacy at 5-years follow-up and depressive symptoms over time, whilst adjusting for potential confounders. To assess different trajectories of depressive symptoms over time, four categories were created; 1. *no depressive symptoms* (*n* = 667)*,* 2. *resolved depressive symptoms* (*n* = 165), 3. *new depressive symptoms* (*n* = 189), and 4. *persistent depressive symptoms* (*n* = 243).

**Results:**

There was very strong evidence of a difference in total healthy eating self-efficacy at follow-up between the four depressive symptoms trajectory categories (F(3,235) = 7.06,*p* < .0001), after adjusting for potential confounders. Pairwise comparisons indicated strong evidence of higher healthy eating self-efficacy among individuals with *no depressive symptoms* compared to individuals with *persistent depressive symptoms* (B = 1.97[95%CI: 0.60,3.33],*p* = .005). Similarly, there was evidence of higher healthy eating self-efficacy in individuals with *resolved depressive symptoms* than those with *persistent depressive symptoms* (B = 1.95[95%CI: 0.18,3.72],*p* = .031).

**Conclusions:**

This study provides new insights demonstrating differences in total healthy eating self-efficacy at 5-year follow-up according to trajectory of depressive symptoms over time. Future interventions should focus on strategies that enhance self-efficacy among individuals with or at risk of depressive symptoms for supporting healthier dietary practices, which in turn, may contribute to reducing the highly burdensome mental health condition.

## Background

Depression is a common illness, with more than 264 million people of all ages affected globally [1]. It is a leading cause of disability worldwide, and the burden of depression is on the rise [2]. There are effective psychological and pharmacological treatments for moderate and severe depression, yet non-adherence to antidepressant treatment is very common [3, 4]. Lifestyle options are commonly more well-accepted than pharmacological treatment approaches by patients [5], and there is growing evidence that diet is associated with both depressive symptoms and clinical depression [6]. Typically, it is assumed that diet and depression are related through biological mechanisms including inflammation, oxidative stress, gastro-intestinal microbiota and neurotrophic factors [7, 8]. However, it is also plausible that depression impacts diet, for example by impairing the personal drivers of healthy eating behaviors [9]. One of the most consistently observed personal drivers of healthy eating is self-efficacy. Self-efficacy refers to a person’s perceived beliefs about their capabilities to produce a desired action or exercise influence over events that affect their lives [10] e.g., an individuals’ confidence in their ability to make the behavior changes necessary to achieving their goals [10]. Self-efficacy beliefs affect life choices, level of motivation, quality of functioning, resilience to adversity and vulnerability to stress and depression [10, 11]. Dietary/healthy eating self-efficacy refers to the perceived capability to make healthy food choices, even when faced with potential barriers [12]. Increasing self-efficacy may lead to healthy eating behavior change. For example, higher dietary self-efficacy has been shown to be associated with higher intakes of fruit and vegetables [13], calcium-rich foods [14] and lower intakes of fat [15]. Conversely, lower self-efficacy beliefs are related to unhealthy food intake [16].

Studies examining the relationship between general self-efficacy and depression have found that individuals with more serious symptoms of depression at baseline report poorer general self-efficacy at follow-up [17]. However, few studies [17–19] have assessed associations of depression with healthy eating self-efficacy, and fewer have examined this over time. For example, a secondary data analysis (using cross-sectional baseline data) of 743 women who were participating in a workplace wellness RCT, found that depressive symptoms were associated with decreased healthy eating self-efficacy, as well as more frequent emotional eating [18]. Additionally, in a cohort study of 18 year-old Australian men (*n* = 301) and women (*n* = 282), depression scores were inversely cross-sectionally associated with confidence in being able to stick to a healthy diet [19]. Finally, greater depressive symptoms were correlated with reports of poorer dietary intent and choice, less perceived support for healthy eating, and poorer self-efficacy for diet in 198 urban youth at risk for developing type 2 diabetes mellitus [20].

While these limited cross-sectional findings suggest a link between depressive symptoms and healthy eating self-efficacy, further research using prospective study designs is required to establish the temporal nature of associations identified. Hence, the aim of this study was to explore the associations of depressive symptoms with healthy eating self-efficacy over time. This is important because if depressive symptoms are linked to reduced self-efficacy, dietary interventions for people with, or at risk of depression, may need to focus intensively on enhancing self-efficacy for healthy eating.

More specifically, this study explores the association of depressive symptoms with healthy eating self-efficacy over time (from baseline (T1) to 5 years follow-up (T3)) amongst women living in socioeconomically disadvantaged neighborhoods in Australia. This is an important sample in which to assess the associations of depressive symptoms with healthy eating self-efficacy, given that women are at significantly greater risk of depression than men [21], and that both depression and unhealthy eating behaviors are more prevalent in socioeconomically disadvantaged neighborhoods [22, 23].

## Methods

### Study sample

Data was drawn from the Resilience for Eating and Activity Despite Inequality (READI) longitudinal study, a prospective cohort study of 4349 women aged 18–46 years recruited randomly using the electoral roll from 80 socio-economically disadvantaged urban and rural neighborhoods of Victoria, Australia. The aims of the READI study were to investigate pathways (personal, social and structural) by which socio-economic disadvantage influences lifestyle choices associated with obesity risk (physical inactivity, poor dietary choices), and to explore mechanisms underlying ‘resilience’ to obesity risk in socioeconomically disadvantaged women and children. Detailed methods are provided elsewhere [24]. The study was approved by the Deakin University Human Research Ethics Committee. Women provided written, informed consent to participate.

**Fig. 1:**
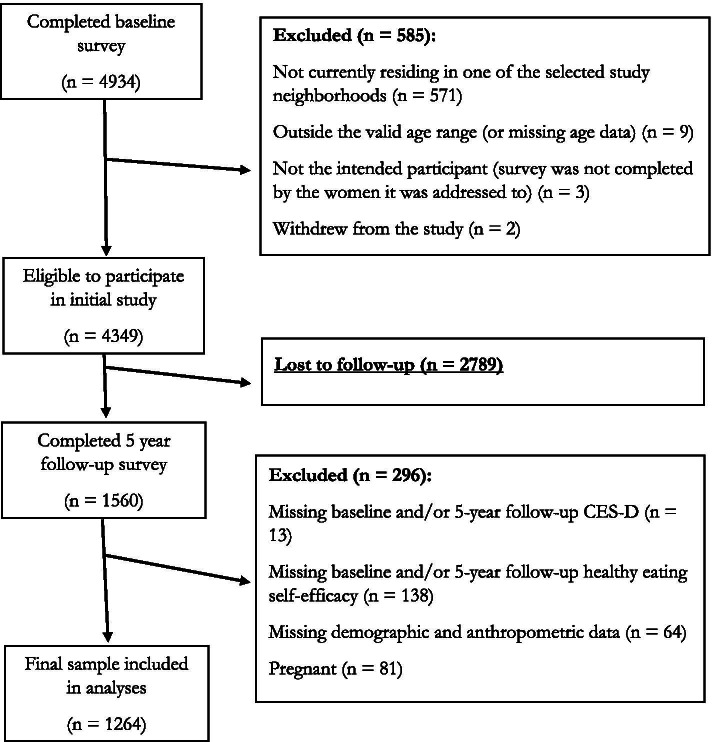
Flowchart of inclusion and exclusion into the study

In late 2007 and early 2008, a total of 4934 women (45% response rate) completed a mailed survey at baseline. Of those that responded, 585 women were excluded for reasons specified in Fig. 1. Thus, 4349 women remained who were eligible to participate (e.g., resided in ‘READI’ neighborhoods, were within the valid age range (between 18 and 45 years), and did not have missing data). Of this cohort, those who consented to further follow-up and remained eligible (*n* = 3019 women) were re-contacted to complete a follow-up survey 3 years after the baseline survey (2010–11). This survey was completed by 1913 women (44% of initial cohort). A further follow-up survey 5 years after the baseline survey (2012–13) was completed by 1560 women (36% of initial cohort).

Women who did not complete questions on depressive symptoms, healthy eating self-efficacy score, or who had missing demographic or anthropometric data at baseline or 5-year follow-up were excluded. Additionally, because dietary behaviors typically alter in pregnancy [25], and are therefore not reflective of habitual intake, pregnant women (at any time point) were not included. Thus, of the total possible baseline and 5 year follow-up completers (*n* = 1560), 1264 women remained in the study for analyses (29% of initial cohort). Refer to Fig. 1 for flowchart of inclusion and exclusion into the study.

### Healthy eating self-efficacy measures

At baseline and 5 years follow-up, a 17-item self-efficacy scale [25] was used to assess individuals’ confidence in performing a number of food related activities, such as procuring (“How confident are you that you could shop regularly for healthy nutritious foods over the next year”), preparing (“…prepare/cook healthy nutritious foods”), and eating healthy foods (e.g., “…eat enough fruit and vegetables for good health”), and limiting consumption of less healthy options (e.g., “limit your fast food consumption to once a week or less”). Additionally, the scale assessed individuals’ confidence for making healthy food choices (“stick to low-fat healthy foods”) according to social or environmental factors (e.g., “when eating with friends or co-workers”; “when you are eating out”; “when you are alone and there is no one to watch you”; “when there are high-fat foods available”), and emotional situations (e.g., “when you feel depressed, bored or tense”; “when you are craving less healthy foods”; “when you feel too tired or lazy”). This scale was adapted from an existing self-efficacy scale for health-related diet behaviors developed by Sallis et al., which has demonstrated internal consistency reliabilities and criterion-related validity [25]. Responses were provided via a 5-point Likert scale ranging from 1 “not at all to 5 “extremely confident”. In the present study, three items were omitted from analysis a priori, because they were either not applicable to a broad number of women (“stick to low-fat healthy foods even when you are eating at work/ place of study”) or they were not well-aligned with the overarching themes relating to procuring, preparing, social / environment factors, or emotional situations (e.g., “not eat meals while watching TV”, “not eat snacks while watching TV”). In addition to considering the individual items of the scale, in order to gain insights into important specific components of self-efficacy, we also calculated a single composite score by summing responses across 14 items. The total score ranged from 14 to 70, with a higher score indicating greater confidence. Cronbach alpha coefficients for the total healthy eating self-efficacy scale were 0.95 at both baseline and 5 years follow-up, indicating high internal consistency.

### Depressive symptoms measure

Depressive symptoms were assessed using the 10-item Center for Epidemiologic Studies Depression Scale (CES-D) [26] at baseline and 5 years follow-up. This scale is a commonly-used self-reported measure of depressive symptoms with good retest reliability and predictive validity compared with the original 20-item version [26–30]. Responses were reported using a four-point Likert scale ranging from ‘rarely’ (scored 0) to ‘most of the time’ (scored 3). The total score was derived by calculating the sum of 10 items (possible scores range from 0 to 30), with higher scores indicating a higher presence of depressive symptoms. Depressive symptoms were presented as a dichotomous variable with a CES-D score of ≥10 used to define risk of heightened depressive symptoms. This cut-off value has been shown to produce good test-retest reliability [26, 28]. Additionally, in order to assess different trajectories of depressive symptoms over time, the following four categories were created; Group 1 *no depressive symptoms* (CES-D < 10 at T1 and T3), Group 2 *resolved depressive symptoms* (CES-D ≥ 10 at T1, but CES < 10 at T3), Group 3 *new depressive symptoms* (CES < 10 at T1, but CES-D ≥ 10 at T3), and Group 4 *persistent depressive symptoms* (CES-D ≥ 10 at T1 and T3).

### Statistical analysis

Data was analysed using the Statistical Package for the Social Sciences (SPSS) version 26 [31]. *P*-values < .001 were deemed to indicate very strong evidence (against the null hypothesis), *p*-values < .01 indicated strong evidence, *p*-values < .05 indicated some evidence, *p*-values < .1 indicated little evidence, and *p*-values ≥ .1 indicated insufficient evidence [32]. Baseline characteristics of the final eligible sub-sample included in this analysis (*n* = 1264) were compared to those of the remaining (excluded) READI sample (*n* = 3085). When comparing the two groups Chi-square tests were used for categorical variables and independent samples t-tests were performed for continuous variables.

#### Longitudinal associations between healthy eating self-efficacy and depressive symptoms

Linear mixed models, with random intercepts for suburb of residence, were performed to explore the relationships between 1. Total healthy eating self-efficacy score and individual items at 5-years follow-up (continuous, dependant variables) according to baseline depression risk (categorical, independent variable), and 2. Total healthy eating self-efficacy score at 5 years follow-up (continuous, dependant variable) and depressive symptoms over time (categorical, independent variable). Unadjusted models were fitted as well as models adjusting for the baseline level of the healthy eating self-efficacy outcome plus a number of potential confounders selected a-priori based on existing evidence that these variables are likely to play a role in diet / self-efficacy for healthy eating and depressive symptoms. All confounders were measured at baseline, and were as follows: age (continuous); highest education level (low (less than high school), medium (high school/trade/diploma), high (tertiary)); country of birth (Australia, overseas); employment status (full-time, part-time, not currently employed); relationship status (married/de facto, separated/ divorced/ widowed, never married); location of residence (urban, rural); smoking status (never smoked, former smoker, occasional smoker, regular smoker); BMI (based on self-reported height and weight), and leisure-time physical activity, assessed using the International Physical Activity Questionnaire (IPAQ) [33]. Total leisure time physical activity represented the weekly sum of the self-reported number of minutes spent in vigorous- and moderate- intensity leisure-time physical, and walking for leisure.

An additional analysis was conducted to examine associations between concurrent changes in total healthy eating self-efficacy (continuous dependent variable) and depressive symptoms (continuous independent variable) over time. Fixed-effects models, with repeated measures across participants using data from all three timepoints (baseline, 3-year follow-up, and 5-year follow-up), were fitted with cluster-robust standard errors [34] to account for clustering of participants within suburbs. In this model, any participants with data for two or more time points contributed to the effect estimate. As participants serve as their own control in fixed-effects models, there is no confounding by time-invariant factors, however as confounding can occur for time-varying factors [35], participant age and BMI were included in the model as time-varying covariates.

### Sensitivity analysis

It was considered that some participants might have a relatively small increase or decrease (e.g., one or two points) in the depressive symptoms score from baseline to 5-year follow-up, which may be considered a clinically insignificant difference and plausibly due to measurement error, and yet have crossed over from low (CESD-10 score of < 10) to high (≥10) risk of depression (or vice-versa) between time points. As a sensitivity analysis, the longitudinal linear mixed model testing the association between total healthy eating self-efficacy score at 5-year follow up and depressive symptoms over time was re-run, excluding any individuals who had a CES-D score change of ±1–2 points, crossed over the cut-off / threshold value for depressive symptoms (CES-D ≥ or < 10), and were classified as either *new depressive symptoms* or *resolved depressive symptoms* in the primary analysis*.*

## Results

Comparing the final eligible sub-sample (*n* = 1264) and the excluded READI sub-sample (*n* = 3085), the eligible sub-sample participants had lower mean CES-D scores at baseline (mean = 7.9 [SD = 5.4] vs. mean = 8.5 [SD = 5.6])and were less likely to be at high risk for depression, were older, and were more likely to be Australian born, married, reside in a rural setting, and have completed tertiary education (Table 1). Additionally, the eligible sub-sample participants were less likely to be unemployed or smoke compared to the excluded sub-sample. There was little evidence of group differences for healthy eating self-efficacy, BMI, or leisure time physical activity.

**Table 1:** Baseline characteristics of the final eligible sub-sample included in this analysis compared to the excluded READI sample

	Included eligible sub-sample (*n* = 1264)	Excluded READI sub-sample (*n* = 3085)	*p*-value
Depression (CES-D), mean (SD)	7.9 (5.4)	8.5 (5.6)	<.0001
High risk for depression (CES-D ≥ 10), % (n)	32.3 (408)	38.6 (1167)	<.001
Total healthy eating self-efficacy, mean (SD)	44.8 (10.9)	44.2 (11.3)	.072
Age, mean (SD)	36.8 (7.5)	33.5 (8.2)	<.0001
Highest education level, % (n)			
Low (less than high school)	22.6 (285)	21.9 (661)	.001
Medium (high school/trade/diploma)	47.7 (600)	53.5 (1616)	
High (tertiary)	29.7 (374)	24.7 (746)	
Born in Australia, % (n)	91.9 (1162)	87.7 (2689)	<.0001
Employment status, % (n)			
Full-time	36.2 (451)	38.9 (1162)	<.0001
Part-time	34.1 (425)	27.5 (820)	
Not currently employed	29.6 (369)	33.6 (1003)	
Relationship status, % (n)			
Married/de facto	71.4 (902)	63.0 (1927)	<.0001
Separated/ divorced/ widowed	7.8 (99)	8.9 (271)	
Never married	20.7 (262)	28.1 (861)	
Area of residence, % (n)			
Urban	41.1 (520)	48.5 (1496)	<.0001
Rural	58.9 (744)	51.5 (1589)	
Smoking status, % (n)			
Never smoked	51.7 (654)	49.6 (1530)	.001
Former smoker	27.1 (342)	23.5 (724)	
Current smoker	21.2 (268)	26.9 (828)	
BMI categorised^a^, % (n)			
Under/healthy weight	53.8 (655)	52.3 (1498)	.652
Overweight	25.1 (306)	25.5 (731)	
Obese	21.1 (257)	22.2 (635)	
Leisure time physical activity (total minutes per week), mean (SD)	210.1 (269.7)	211.8 (323.4)	.856

^a^BMI categorised as under/healthy weight, overweight, obese according to WHO cut-points [36]

At baseline, the mean value for the total healthy eating self-efficacy score was 44.9 (SD ± 10.9), with a large range in scores from 16 to 70, and at 5-years follow-up the mean score was 45.6 (SD ± 11.4; range 14 to 70). Table 2 details the mean depressive symptom scores for the four depressive symptom trajectory categories.

**Table 2:** Mean (SD) depressive symptom scores for each depressive symptoms trajectory category at baseline and follow-up

Depressive symptoms trajectory category		Baseline	5-Year Follow-Up
	n	CES-D Mean (SD)	CES-D Mean (SD)
*No depressive symptoms*	667	4.5 (2.5)	4.5 (2.4)
*Resolved depressive symptoms*	165	13.3 (3.4)	6.0 (2.3)
*New depressive symptoms*	189	5.9 (2.3)	13.7 (3.8)
*Persistent depressive symptoms*	243	14.8 (4.3)	15.1 (4.2)

### Healthy eating self-efficacy at 5-year follow-up according to baseline depression risk

In the adjusted model, there was virtually no evidence of a difference in total healthy eating self-efficacy at 5-year follow-up among individuals with baseline low risk for depression (CES-D < 10) compared to individuals with high risk for depression (CES-D ≥ 10) (Table 3). For the individual self-efficacy items, there was evidence of higher healthy eating self-efficacy at 5-year follow-up for shopping regularly for healthy nutritious foods (β = 0.15 [95% CI:0.05,0.26], *p* = .004), preparing/cooking healthy nutritious foods (β = 0.11 [95% CI:0.01,0.22], *p* = .030), and eating enough vegetables for good health (β = 0.11 [95% CI:0.01,0.21], *p* = .038), among women with low risk for depression at baseline compared to high risk individuals (adjusted models). There was little to no evidence of differences between the two groups for the remaining healthy eating self-efficacy items.

**Table 3:** Healthy eating self-efficacy scores at 5-year follow-up according to baseline depression risk

		Low risk for depression (CES-D < 10) *n* = 856	High risk for depression (CES-D ≥ 10) *n* = 408	Estimated mean difference	
	Model	M (SD)	M (SD)	B (95% CI)	*p*-value
Total healthy eating self-efficacy	Unadjusted	45.7 (10.7)	43.3 (12.4)	3.41 (2.08 to 4.74)	<.0001
	Adjusted			0.45 (−0.62 to 1.51)	.411
Healthy eating self-efficacy item:					
**Procuring**					
1. Shop regularly for healthy nutritious foods over the next year	Unadjusted	3.9 (0.9)	3.5 (1.1)	0.35 (0.24 to 0.47)	<.0001
	Adjusted			0.15 (0.05 to 0.26)	.004
**Preparing**					
2. Prepare/cook healthy nutritious foods over the next year	Unadjusted	3.8 (0.9)	3.5 (1.1)	0.33 (0.21 to 0.44)	<.0001
	Adjusted			0.11 (0.01 to 0.22)	.030
**Eating**					
3. Stick to eating healthy nutritious foods over the next year	Unadjusted	3.5 (1.0)	3.2 (1.1)	0.27 (0.14 to 0.39)	<.0001
	Adjusted			0.03 (−0.08 to 0.14)	.569
4. Eat enough fruit for good health over the next year	Unadjusted	3.5 (1.1)	3.2 (1.2)	0.31 (0.18 to 0.44)	<.0001
	Adjusted			0.09 (−0.03 to 0.21)	.146
5. Eat enough vegetables for good health over the next year	Unadjusted	3.9 (0.9)	3.6 (1.0)	0.31 (0.20 to 0.41)	<.0001
	Adjusted			0.11 (0.01 to 0.21)	.038
6. Limit your fast food consumption to once a week or less over the next year	Unadjusted	4.1 (0.9)	4.0 (1.0)	0.18 (0.07 to 0.30)	.001
	Adjusted			0.00 (−0.11 to 0.11)	.978
7. Eat a low-fat diet over the next year	Unadjusted	3.3 (1.0)	3.1 (1.1)	0.15 (0.02 to 0.28)	.020
	Adjusted			−0.07 (−0.19 to 0.04)	.196
**Emotional situations**					
8. Stick to low-fat healthy foods even when you feel depressed, bored or tense	Unadjusted	2.8 (1.1)	2.6 (1.2)	0.24 (0.11 to 0.38)	<.0001
	Adjusted			0.01 (−0.12 to 0.13)	.932
9. Stick to low-fat healthy foods even when you feel too tired or lazy to prepare something healthy	Unadjusted	2.9 (1.0)	2.6 (1.1)	0.24 (0.12 to 0.36)	<.0001
	Adjusted			0.01 (−0.09 to 0.12)	.793
10. Stick to low-fat healthy foods even when you are craving less healthy foods	Unadjusted	2.6 (1.0)	2.5 (1.0)	0.14 (0.03 to 0.26)	.016
	Adjusted			−0.05 (−0.16 to 0.06)	.350
**Social or environmental factors**					
11. Stick to low-fat healthy foods when you are eating out	Unadjusted	2.8 (1.0)	2.6 (1.1)	0.15 (0.03 to 0.28)	.016
	Adjusted			−0.03 (−0.15 to 0.08)	.569
12. Stick to low-fat healthy foods even when there are high-fat foods available	Unadjusted	3.1 (1.0)	2.9 (1.1)	0.23 (0.10 to 0.35)	<.0001
	Adjusted			0.00 (−0.11 to 0.11)	.965
13. Stick to low-fat healthy foods even when eating with friends or co-workers	Unadjusted	3.2 (1.0)	3.0 (1.1)	0.22 (0.10 to 0.34)	<.0001
	Adjusted			0.01 (−0.10 to 0.12)	.866
14. Stick to low-fat healthy foods even when you are alone and there is no one to watch you	Unadjusted	3.3 (1.1)	3.0 (1.2)	0.29 (0.16 to 0.42)	<.0001
	Adjusted			0.08 (−0.04 to 0.20)	.197

Unadjusted model. Estimated mean differences from mixed-effects linear models with random intercepts for suburbs.

Adjusted model. Estimated mean differences from mixed-effects linear models with random intercepts for suburbs and adjusted for baseline healthy eating self-efficacy, age, education, country of birth, employment, marital status, area of residence, smoking status, leisure time physical activity, and BMI.

### Healthy eating self-efficacy and depressive symptoms over time

There was very strong evidence of a difference in total healthy eating self-efficacy at 5-year follow-up between the four depressive symptoms trajectory categories (F(3,235) = 7.06, *p* < .0001), while adjusting for potential confounders. Pairwise comparisons indicated strong evidence of higher healthy eating self-efficacy among individuals with *no depressive symptoms* (*n* = 667) compared to individuals with *persistent depressive symptoms* (*n* = 243) (estimated mean difference B = 1.97 [95% CI: 0.60, 3.33], *p* = .005). Similarly, there was evidence of higher healthy eating self-efficacy in individuals with *resolved depressive symptoms* (*n* = 165) than those with *persistent depressive symptoms* (B = 1.95 [95% CI: 0.18, 3.72], *p* = .031).

In the fixed-effects model, there was very strong evidence for an association between concurrent change in depressive symptoms and total healthy eating self-efficacy (B = -0.15 [95% CI: − 0.23, − 0.07], *p* < .0005), indicating that for a one-unit within-individual change in CES-D score there was an average decrease in healthy eating self-efficacy score of 0.15.

### Sensitivity analysis

Seventeen individuals with *resolved depressive symptoms* (10%, total *n* = 165) and 15 individuals with *new depressive symptoms* (8%, total *n* = 189) reported a CES-D score change of ±1–2 points, and crossed over the cut-off / threshold value for depressive symptoms (CES-D ≥ or < 10). As a sensitivity analysis, the linear mixed model examining associations between total healthy eating self-efficacy score and depressive symptoms trajectory category was re-run excluding these 32 individuals, and the results remained generally consistent with the primary analysis (data not shown).

## Discussion

This is one of the first studies to explore the relationship between self-efficacy for healthy eating and depressive symptoms over time. It provides new insights demonstrating differences in total healthy eating self-efficacy at 5-year follow-up according to trajectory of depressive symptoms over time. Specifically, healthy eating self-efficacy scores were found to be higher among individuals who reported *no depressive symptoms over time*, and among individuals with *resolved depressive symptoms* at 5-year follow-up, compared to individuals with *persistent depressive symptoms*. Women with baseline low risk for depression had higher healthy eating self-efficacy at 5-year follow-up for shopping, and preparing/cooking healthy nutritious foods, and eating enough vegetables for good health, compared to those at high risk for depression. Additionally, the fixed-effects model demonstrated evidence of an association, such that as depressive symptoms increased over time, self-efficacy for healthy eating decreased (and vice-versa).

Self-efficacy is defined as an individuals’ confidence in their ability to make the behavior changes necessary to achieving their goals [11], and has been associated with the adoption of and engagement in many nutrition and health behaviors, including changes in weight and weight-related behaviors [37]. Hence, it is worth considering that the association between healthy eating self-efficacy and depression may be bidirectional. For example, enhancing self-efficacy has been viewed as an important strategy for promoting improvements in dietary intake [37], because higher self-efficacy has been associated with a healthy dietary pattern [16] [38], which in turn may be associated with reduced risk of depression [39].On the other hand, self-efficacy is commonly diminished in depressed individuals [40], and the varying trajectories of mental illness means motivation and self-efficacy can be impaired or fluctuate, which in turn may lead to poor eating behaviors [18] and poor adherence to dietary recommendations [41]. This is consistent with findings from our study, where individuals at high risk for depression had lower healthy eating self-efficacy for procuring, preparing, cooking and eating healthy foods.

To further compound this, common side effects of lowered mood and depression include changes in appetite, which can involve either a reduced interest in food or an increase appetite with cravings for less healthy choices like sweet, salty and fatty foods [42–44]. Depression can also be commonly associated with fatigue and apathy, which may impact on an individual’s motivation to engage in healthy dietary habits [45], and reduced energy for grocery shopping, meal preparation, cooking, and clean-up [43, 46–49]. As a result of decreased concentration, decreased mental endurance and slowed thinking [49] individuals may also find learning new recipes or developing cooking skills challenging [47, 48]. Our earlier analysis of adherence to the Australian Dietary Guidelines amongst the READI cohort, similarly showed that baseline diet quality was poorer amongst individuals with depressive symptoms at follow-up, compared to individuals without depressive symptoms [50]. When considering that a healthy dietary pattern (high in vegetables, fruits, whole grains, fish, and legumes) is associated with a decreased risk of depression, [6, 51], and that an unhealthy dietary pattern (high in processed foods, refined grains, and sweets) may increase the risk of depression [6, 51], the current findings suggest the importance of the development of strategies to promote self-efficacy among individuals with /or at risk of depressive symptoms.

Goal setting, problem solving (e.g., meal planning, recipe modification), and provision of personalised feedback can help individuals increase their self-efficacy for changes in their eating behavior [37, 52]. Furthermore, learning basic cooking skills and hands-on learning of healthy recipes is empowering and has previously been associated with healthier food choices [52]. This relates to food literacy, an important tool needed to support a healthy lifelong relationship with food – it empowers individuals to achieve optimal diet quality through skills and behaviors required to plan, manage, select, prepare, and eat food to meet needs [53]. Motivational interviewing and health coaching, which foster good communication and therapeutic relationships, are likely integral intervention components for improving self-efficacy and dietary adherence [54]. Future studies could test the efficacy of all of these components for promoting enhanced dietary self-efficacy and healthy eating amongst people at risk of or experiencing depression.

### Limitations and strengths

A potential study limitation is that only 29% of the original READI sample were eligible for analysis in the present study. However, this attrition rate of 71% at 5-year follow-up is noteworthy when considering that other longitudinal community-based nutrition research among women report considerable range in attrition rates from 43 to 75% [55]. The proportion of women reporting depressive symptoms (32%, *n* = 408 out of 1264) in our cohort were higher than that of the Australian population-based National Health Survey [56], although the latter was based on an assessment of women’s self-reported “depression or feelings of depression” rather than on a multi-item symptom scale, which could at least partly account for the difference. Moreover, there was a reported difference in the % at high risk for depression among the included (32.3%) and excluded (38.6%) participants in our study, which may limit generalisability. The READI study sample was recruited from disadvantaged neighborhoods and hence generalisability to other samples may be limited; although our eligible sub-sample were of a slightly higher socioeconomic position than the remaining READI sample. Pregnant women were excluded from this study, which may also limit generalisability of findings. Future studies should be conducted in this population group, as perinatal depression (from conception to the end of the first year of a child’s life) has a high global prevalence [57], poses significant costs to individuals, families and society, and has shown to be associated with poorer dietary quality [58].

Depressive symptoms were assessed using the CES-D, a commonly-used self-reported measure, with a cut-off score (e.g., 10 or greater) that aids in identifying individuals at risk for clinical depression, with good sensitivity, specificity and high internal consistency [26, 59], and is able to identify individuals with significant depression with good precision [60]. In this analysis, the CES-D was presented as a categorical variable, rather than a continuous variable, which is useful for comparing low/high risk groups, but may have resulted in some women being categorised as changing trajectory on the basis of a very small change in score. To address this, we conducted a sensitivity analysis, and the results remained generally consistent with those of the primary analysis. A major strength of this study is the use of longitudinal data to explore the associations between depressive symptoms over time with healthy eating self-efficacy, which to our knowledge has not been performed before. Finally, due to the large sample size we were also able to control for relevant confounders.

## Conclusion

This study provides new insights demonstrating that depressive symptoms, as well as symptom trajectory over time, are associated with self-efficacy for healthy eating among women living in socioeconomically disadvantaged neighborhoods in Australia. Future interventions should focus on strategies that enhance self-efficacy among individuals with or at risk of depressive symptoms for supporting healthier dietary practices and improving dietary adherence. This in turn, may help improve dietary intakes and contribute to reducing this highly burdensome mental health condition.

## Data Availability

The datasets used and/or analysed during the current study are available from the last author (KB) on reasonable request.

## Abbreviations

CES-D: Center for Epidemiologic Studies Depression Scale
READI: Resilience for Eating and Activity Despite Inequality
SPSS: Statistical Package for the Social Sciences
